# Comparison of ablation outcomes of the second ablation procedure for recurrent atrial fibrillation using an ultra‐high‐resolution mapping system and conventional mappings system

**DOI:** 10.1002/clc.23248

**Published:** 2019-08-12

**Authors:** Masaharu Masuda, Mitsutoshi Asai, Osamu Iida, Shin Okamoto, Takayuki Ishihara, Kiyonori Nanto, Takashi Kanda, Takuya Tsujimura, Yasuhiro Matsuda, Shota Okuno, Aki Tsuji, Toshiaki Mano

**Affiliations:** ^1^ Kansai Rosai Hospital Cardiovascular Center Amagasaki Hyogo Japan

**Keywords:** atrial fibrillation, repeat ablation procedure, ultra‐high‐resolution mapping system

## Abstract

**Background:**

The utility of an ultra‐high‐resolution electroanatomical mapping system (UHR‐EAM, Rhythmia) for repeat atrial fibrillation (AF) ablation has not been evaluated.

**Hypothesis:**

A second AF ablation procedure performed using UHR‐EAM may demonstrate different outcomes compared with that using a conventional electroanatomical mapping system (C‐EAM, CARTO3).

**Method:**

This observational study enrolled consecutive patients who underwent a second AF ablation procedure using UHR‐EAM (n = 103) and C‐EAM (n = 153). The second ablation procedure included re‐isolation of reconnected pulmonary veins (PVs) and elimination of clinical or induced non‐PV AF triggers and atrial tachycardia (AT). Other empirical ablations were additionally conducted at the discretion of the operators.

**Results:**

Re‐isolation of PVs was achieved in 196 patients who had ≥1 left atrial‐PV reconnection. The elimination rate of AT was higher in the UHR‐EAM group than the C‐EAM group (87% vs 65%, *P* = .040), while that of non‐PV AF triggers was similar (63% vs 63%, *P* = 1.00). The UHR‐EAM demonstrated shorter radiofrequency application time (21.8 ± 16.8 vs 28.0 ± 21.3 minutes, *P* = .017), but longer fluoroscopic time (26.2 ± 12.6 vs 21.4 ± 9.3 minutes, *P* = .0001). No severe complication developed. The total 1‐year AF/AT‐free survival rates were similar between the two groups (off AADs, 59.2% vs 56.2%, *P* = .62; on AADs, 65.0% vs 69.3%, *P* = .49).

**Conclusion:**

The efficacy and safety outcomes of repeat AF ablation using UHR‐EAM was comparable to those using C‐EAM.

## 1 INTRODUCTION

Catheter ablation of atrial fibrillation (AF) has become common practice following advancements in electroanatomical mapping systems (EAMs). While a contemporary world‐wide expert consensus recommends pulmonary vein (PV) isolation as an essential procedure of AF ablation, AF recurrence remains a considerable problem.^1^, ^2^ In contrast to the initial ablation procedure, ablation methods in the repeat procedure are often varied, and can include re‐isolation of reconnected PVs, elimination of atrial tachycardias (ATs) and non‐PV AF‐triggering ectopies, and other substrate modifications.

Recently, a new ultra‐high‐resolution EAM (UHR‐EAM; Rhythmia, Boston Scientific, Marlborough [Cambridge], Massachusetts) that allows rapid ultra‐high‐resolution electroanatomical mapping was introduced. Several reports have demonstrated the utility of UHR‐EAM for AF ablation, including PV isolation and modification of extra‐PV arrhythmogenic substrates of various atrial tachyarrhythmias,^3^, ^4^, ^5^, ^6^, ^7^ suggesting that UHR‐EAM may improve rhythm outcomes after AF ablation. The advantages of UHR‐EAM are expected to be even more pronounced in the repeat AF ablation procedure, where the ablation target is often more dependent on the patient's arrhythmogenic substrate, than in the initial ablation procedure. For these reasons, the use of UHR‐EAM in repeat AF ablation is expected to substantially increase.

Here, we compared the efficacy of UHR‐EAM and a conventional EAM (C‐EAM; CARTO 3, Biosense Webster, Diamond Bar, California) in the second AF ablation procedure.

## 2 METHODS

### 2.1 Patients

This observational study enrolled patients who underwent a second AF ablation procedure at Kansai Rosai Hospital from April 2014 to Apr 2018. The procedural outcomes of 103 consecutive patients who received a second AF ablation using UHR‐EAM were compared with those of 153 controls who received a second AF ablation using C‐EAM. The selection of mapping system was at the discretion of the attending physician. Most patients ablated early in the study done with C‐EAM and later patients with UHR‐EAM. This study complied with the Declaration of Helsinki. Written informed consent for the ablation and participation in the study was obtained from all patients, and the protocol was approved by our institutional review board.

### 2.2 Catheter ablation procedure

Electrophysiological studies and catheter ablation were performed by three experienced operators (MM, TK, and AS) with the patient under intravenous sedation with dexmedetomidine. A 6‐Fr decapolar electrode was inserted into the coronary sinus while a second 6‐Fr decapolar electrode was placed in the right atrium. Following transseptal puncture at the fossa ovalis, two long sheaths were introduced into the left atrium. A 64‐pole mini‐basket catheter (Orion, Boston Scientific) and an open‐irrigated ablation catheter with a 3.5‐mm tip (Thermocool Celsius, Biosense Webster) were inserted via the long sheaths for UHR‐EAM. A 20‐pole circular catheter and a 3.5‐mm open‐irrigated ablation catheter with a contact‐force sensor (Thermocool ST/SF, Biosense Webster) were used for C‐EAM. Radiofrequency application was set at 30 W with a maximum temperature of 42°C and irrigation rate of 8 mL/min. Details of ablation with the two mapping systems are provided in Table 1.

**Table 1:** Ablation methods using ultra‐high‐resolution electroanatomical mapping and conventional electroanatomical mapping

	UHR‐EAM	C‐EAM
Ablation of a conduction gap after circumferential PV, SVC isolation or linear ablation	Identify a gap on the propagation map obtained by the basket catheter	Estimate a gap using the signal sequence recorded by the ablation or circular catheters
	Point ablation at the gap	Linear ablation along the previous ablation line covering the gap
Focal AT ablation	Mapping using the mini‐basket catheter	Mapping using the circular catheter or ablation catheter
	Ablation at the earliest activation site	Ablation at the earliest activation site
Macro‐reentrant AT ablation	Mapping using the mini‐basket catheter	Mapping using the circular catheter or ablation catheter
	Linear ablation across AT circuits connecting two non‐conducting tissues for macro‐reentrant AT or point ablation at a slow‐conduction isthmus	Linear ablation across AT circuits connecting two non‐conducting tissues for macro‐reentrant AT
Ablation of non‐PV AF trigger	Mapping using the ablation catheter or the basket catheter	Mapping using the circular catheter or ablation catheter
	Point ablation at the earliest activation site	Point ablation at the earliest activation site
	Circumferential SVC isolation for SVC trigger	Circumferential SVC isolation for SVC trigger
Low‐voltage‐area ablation^a^	Voltage map using the basket catheter during sinus rhythm	Voltage map using the ablation catheter during sinus rhythm
	Low‐voltage areas were defined as areas with bipolar peak‐to‐peak voltage of <0.5 mV	Low‐voltage areas were defined as areas with bipolar peak‐to‐peak voltage of <0.5 mV
	Isolation or homogenization of low‐voltage areas	Isolation or homogenization of low‐voltage areas
Cavo‐tricuspid isthmus ablation^a^	Linear ablation	Linear ablation
Left atrial posterior isolation^a^	Posterior isolation by creating roof and bottom ablation lesions	Posterior isolation by creating roof and bottom ablation lesions

a Low‐voltage‐area ablation, empirical SVC isolation, empirical cavo‐tricuspid isthmus ablation and empirical left atrial posterior isolation were additionally conducted at the discretion of the attending operators.

Abbreviations: AF, atrial fibrillation; AT, atrial tachycardia; C‐EAM, conventional mapping system; PV, pulmonary vein; SVC, superior vena cava; UHR‐EAM, ultra‐high‐resolution electroanatomical mapping system.

**Figure 1:**
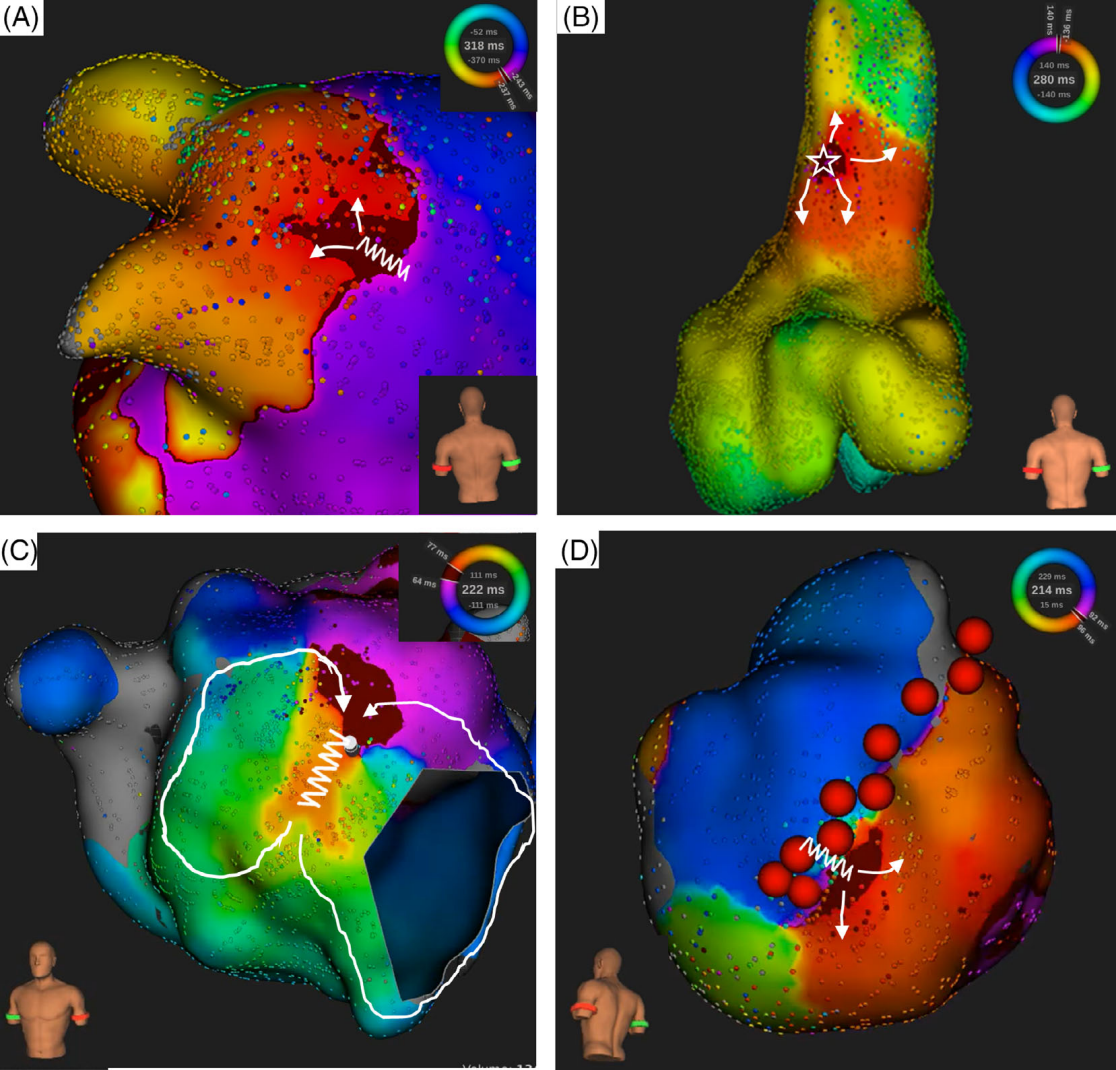
Examples of ablation using an ultra‐high‐resolution electroanatomical mapping (UHR‐EAM) system A, Propagation map showing a left atrium‐left pulmonary vein conduction gap. The gap is clearly visible at the posterior aspect of the initial circumferential pulmonary vein isolation line. A single radiofrequency application at the gap site immediately eliminated the left atrium‐pulmonary vein conduction. B, Propagation map showing a focal atrial tachycardia (AT) originating at the right atrial posterior wall. A point ablation at the earliest activation site (white star) directly terminated the AT. C, Propagation map showing a figure‐of‐eight macro‐reentrant AT with a slow conduction isthmus at the left atrial septum. A point radiofrequency application at the slow conduction isthmus resulted in direct tachycardia termination. D, Propagation map showing a conduction gap after a posterior mitral isthmus linear ablation. Point ablation at the gap achieved immediate bidirectional conduction block. White arrow, wave‐front propagation; white zigzag line, slow conduction zone

Prior to the ablation procedure, we examined the electrical conduction between each PV and the left atrium. If the conduction indicated reconnection, radiofrequency energy was applied at the estimated gap sites. An example map of a left atrium‐PV conduction gap using UHR‐EAM is presented in Figure 1A.

After re‐isolation of reconnected PVs, atrial burst pacing (cycle length 200‐300 msec for 5 seconds) was performed, followed by a high‐dose isoproterenol provocation test (infusion of 5, 10, and 20 μg/min isoproterenol for 2 minutes each) to induce AF or AT. AF‐triggering ectopies or frequent ectopies originating from the superior vena cava (SVC) were eliminated by circumferential SVC isolation. Non‐PV/SVC AF‐triggering ectopies were mapped using the EAM, and ablated at the earliest activation site (Figure 1B). Induced AT was also mapped using the EAM. An ablation targeting the earliest activation site for centrifugal AT or the reentrant circuit for macro‐reentrant AT was performed. Figure 1C shows an example of a propagation map of macro‐reentrant AT and Figure 1D shows an example of conduction gaps along the linear ablation line.

Ablation targeting left atrial low‐voltage areas were additionally conducted at the discretion of the attending operators. Left atrial voltage mapping was performed during sinus rhythm using the 20‐pole circular catheter for C‐EAM and the mini‐basket catheter for UHR‐EAM. Voltage homogenization or electrical isolation of low‐voltage areas was performed by taking account of the preservation of physiological atrial propagation, such as conduction from the anterior left atrium to the appendage.

Empirical ablations, including SVC isolation, isolation of the left atrial posterior wall, and linear ablation of the cavo‐tricuspid isthmus, were additionally conducted at the discretion of the attending operators. Any linear ablations were performed aiming to achieve bidirectional conduction block.

### 2.3 Follow‐up

Patients were followed up every 4 to 8 weeks at the dedicated arrhythmia clinic of our institution for 1 year. Routine electrocardiograms (ECGs) were conducted at each outpatient visit, and 24‐hour ambulatory Holter monitoring was performed 6‐month post‐ablation. When patients experienced symptoms suggestive of an arrhythmia, a surface ECG, ambulatory ECG, and/or cardiac event recording were also conducted. AF/AT recurrence was defined as the occurrence of one the following events from 3 months after the initial ablation (blanking period): (a) AF/AT indicated on a routine or symptom‐triggered ECG during an outpatient visit, or (b) AF/AT of at least 30‐second duration on ambulatory ECG monitoring. Antiarrhythmic drugs (AADs) were not used after the 3‐month blanking period unless AF/AT recurrence was observed. Recurrent AF/AT which was well suppressed using AADs was defined as off‐AAD recurrence, however, not on‐AAD recurrence. Patients complaining of symptoms due to recurrent AF/AT were prescribed AADs at the discretion of the attending physicians.

### 2.4 Statistical analysis

Continuous data are expressed as the mean ± SD or median (interquartile range). Categorical data are presented as absolute values and percentages. Tests for significance were conducted using the unpaired *t* test or a nonparametric test (Mann‐Whiney *U*‐test) for continuous variables and the χ² test or Fishers exact test for categorical variables. AF/AT‐free survival rates were calculated using the Kaplan‐Meier method. Comparison of survival curves between the groups was performed using a two‐sided Mantel‐Haenszel (log‐rank) test. All analyses were performed using commercial software (SPSS version 25.0, SPSS, Inc., Chicago Illinois).

## 3 RESULTS

### 3.1 Patient characteristics

Baseline and procedural characteristics are shown in Table 2. Baseline characteristics, including the CHA_2_D_2_‐VASc score, were similar between the two groups except for a larger left atrial diameter in the UHR‐EAM group than in the C‐EAM group. Created ablation lesions in the initial ablation were comparable between the two groups.

**Table 2:** Patient characteristics

	UHR‐EAM	C‐EAM	
Characteristic	*n* = 103	*n* = 153	*P*
Age, years	68 ± 10	67 ± 9	.65
Female, n (%)	37 (36)	62 (41)	.46
Body mass index, kg/m^2^	24.6 ± 4.8	24.3 ± 5.8	.66
Paroxysmal AF, n (%)	41 (40)	65 (43)	.70
Hypertension, n (%)	51 (50)	89 (59)	.16
Diabetes mellitus, n (%)	19 (19)	18(12)	.15
Heart failure, n (%)	14 (14)	16 (11)	.55
CHA_2_DS_2_‐VASc score	2.2 ± 1.2	2.2 ± 1.4	.87
Echocardiography findings			
Left atrial diameter, mm	42 ± 8	39 ± 7	.020
Left ventricular ejection fraction, %	63 ± 9	65 ± 9	.14
Ablation lesions in the initial session			
PV isolation, n (%)	103 (100)	153 (100)	1.00
SVC isolation, n (%)	2 (2)	3 (2)	1.00
Cavo‐tricuspid isthmus ablation, n (%)	17 (17)	26 (17)	.98
Left atrial linear ablation, n (%)	17 (17)	18 (12)	.29
Ablation lesions in the second session			
Re‐isolation of re‐connected PVs, n (%)	67 (65.0)	109 (71.2)	.29
Isolation of SVC, n (%)	18 (17.5)	24 (15.7)	.73
Cavo‐tricuspid isthmus linear ablation, n (%)	18 (17.5)	24 (15.7)	.73
Ablation of non‐PV/SVC AF‐triggering ectopies, n (%)	16 (15.5)	32 (20.9)	.28
Ablation of AT, n (%)	14 (13.6)	22 (14.4)	1.00
Empirical posterior isolation, n (%)	13 (12.6)	30 (19.6)	.17
Low‐voltage area^a^‐guided ablation, n (%)	36 (35.0)	46 (30.1)	.42
Major complications in the second session			
Bleeding necessitating transfusion, n (%)	0 (0)	0 (0)	
Cardiac tamponade, n (%)	0 (0)	0 (0)	
Esophageal injury, n (%)	0 (0)	0 (0)	
Cerebral infarction/transient ischemic attack, n (%)	0 (0)	0 (0)	
Death, n (%)	0 (0)	0 (0)	

All data indicate mean ± SD.

a Low‐voltage areas were defined as areas with bipolar peak‐to‐peak voltage of <0.5 mV during sinus rhythm. PV, pulmonary vein; SVC, superior vena cava; AT, atrial tachycardia.

### 3.2 Second ablation procedure

**Figure 2:**
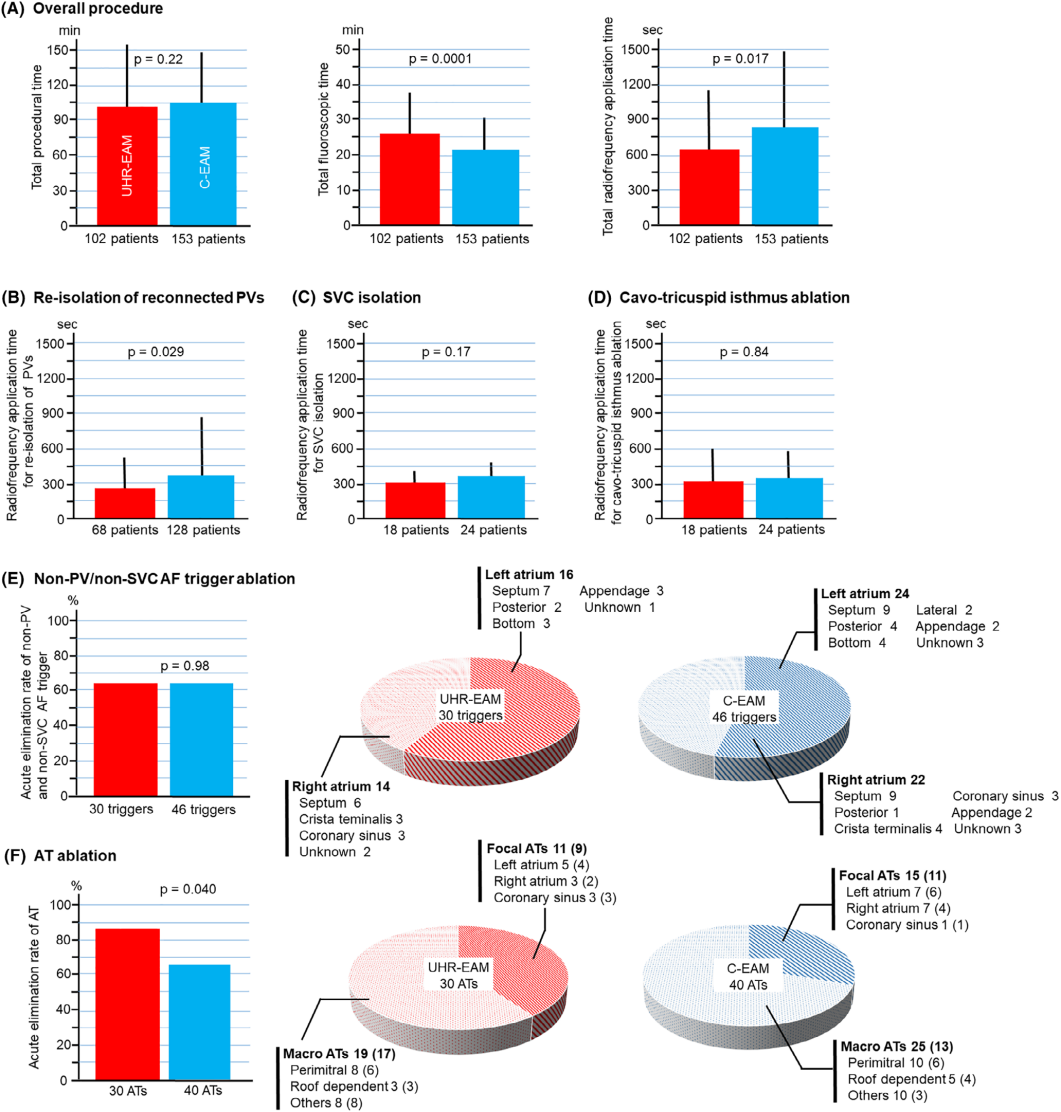
Individual procedural outcomes A, The UHR‐EAM) group demonstrated comparable total procedural time, longer total fluoroscopic time, and lower total radiofrequency application time than the C‐EAM group. B, Radiofrequency application time to re‐isolate reconnected PVs was lower using UHR‐EAM than C‐EAM. C, Radiofrequency application time to isolate SVC was comparable between the two groups. D, Radiofrequency application time to complete bidirectional block line along cavo‐tricuspid isthmus was comparable between the two groups. E, Elimination rate of non‐PV/non‐SVC AF‐triggering ectopies was similar between the two groups. The distribution of the ectopic origins in the two groups is shown in the pie graphs. F, Elimination rate of AT was higher using UHR‐EAM than C‐EAM. The distribution of AT circuits is presented in the pie graphs. The number of ATs that were successfully eliminated by ablation is presented in parentheses. AF, atrial fibrillation; AT, atrial tachycardia; C‐EAM, conventional electroanatomical mapping; PV, pulmonary vein; SVC, superior vena cava; UHR‐EAM, ultra‐high‐resolution electroanatomical mapping;

Procedural characteristics of the second ablation procedure are presented in Table 2. Voltage map during sinus rhythm was created in 88 (86.2%) patients in the UHR group and 138 (90.2%) patients in the C‐EAM group. Creation of ablation lesions was not different between the two groups (Table 2). Total procedural time from sheath insertion to extraction was comparable between the two groups (Figure 2A). In contrast, UHR‐EAM demonstrated 22% lower total radiofrequency application time and 18% longer total fluoroscopic time than C‐EAM.

Individual ablation procedures are compared in Table 2. Re‐isolation of reconnected PVs was completed in all 196 patients who had ≥1 left atrial‐PV reconnection with 28% shorter radiofrequency application time using UHR‐EAM than C‐EAM (Figure 2B). SVC was successfully isolated in all 42 patients who developed frequent or AF‐triggering ectopies from the SVC. Bidirectional conduction block of the cavo‐tricuspid‐isthmus line was also achieved in all 42 patients with induced or clinical common atrial flutter. Procedural outcomes of ablation targeting non‐PV/non‐SVC AF‐triggering ectopies were comparable between the two groups (Figure 2E). Notably, the UHR‐EAM group demonstrated significantly better outcomes for ablation of regular ATs, including macro‐reentrant and focal mechanism ATs (Figure 2E). Left atrial posterior wall isolation was achieved in 38 of 43 patients in whom it was attempted. Ablation targeting low‐voltage areas was performed in 82 patients. No severe complications occurred in either of the two groups.

### 3.3 Long‐term AF recurrence rate

**Figure 3:**
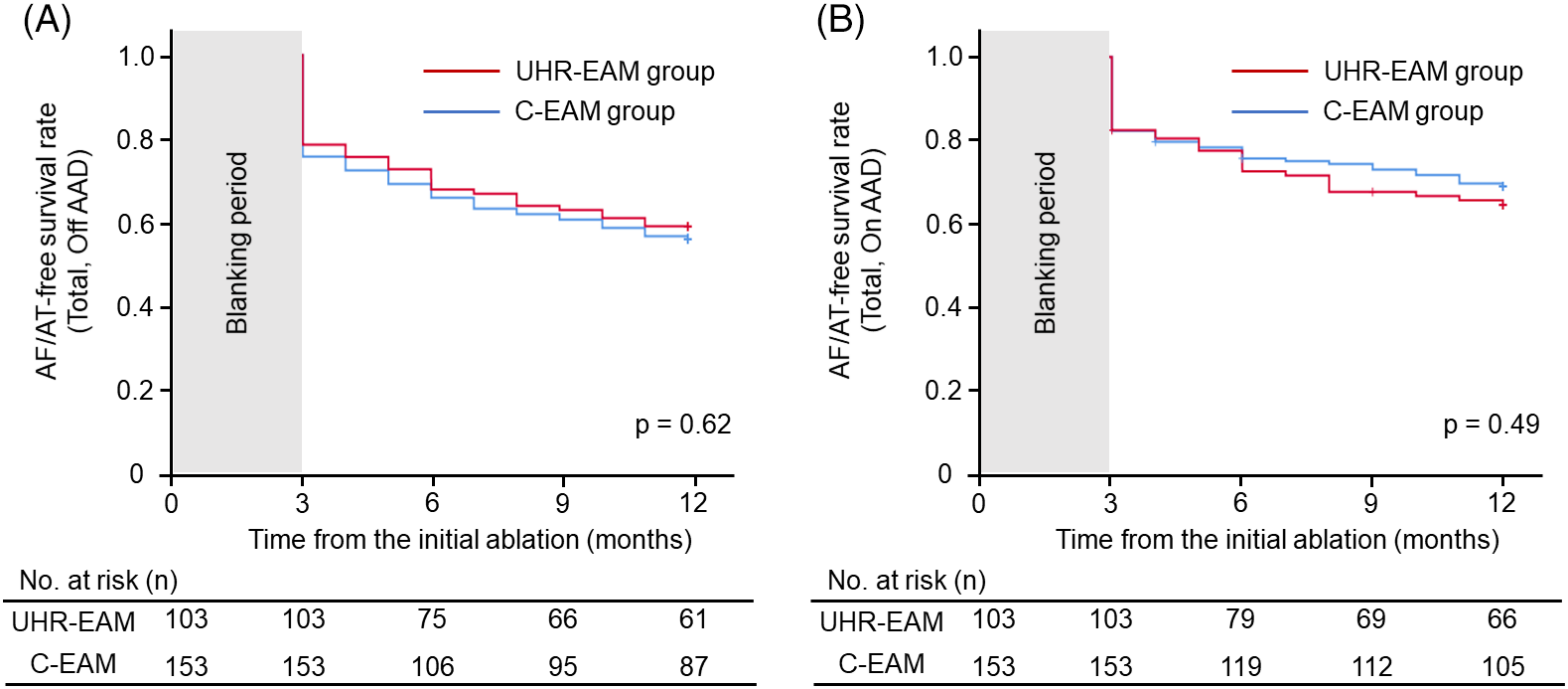
Comparison of the 1‐year AF‐free survival rate between the UHR‐EAM and C‐EAM groups. There were no differences in the total AF recurrence‐free survival rate between the two groups regardless of AAD use. AAD, antiarrhythmic drug; AF, atrial fibrillation; AT, atrial tachycardia; C‐EAM, conventional electroanatomical mapping; PAF, paroxysmal AF; UHR‐EAM, ultra‐high‐resolution electroanatomical mapping

During a 1‐year follow‐up period, 109 of 256 (43%) patients developed AF/AT recurrence without the use of AADs. One patient with AF recurrence in the C‐EAM group died 3 months after the ablation procedure due to obstructive ileus, which was not related to the ablation procedure. There were no differences in the total AF/AT recurrence‐free survival rate between the UHR‐EAM and C‐EAM groups (off AADs, 59.2% vs 56.2%, *P* = .62; on AADs, 65.0% vs 69.3%, *P* = .49; Figure 3). Similarly, the AF/AT‐free survival rate was comparable among patients with paroxysmal AF (off AADs, 68.3% vs 70.8%, *P* = .83; on AADs, 78.0% vs 80.0%, *P* = .81), and non‐paroxysmal AF (off AADs, 53.2% vs 45.5%, *P* = .41; on AADs, 56.5% vs 61.4%, *P* = .61). There was no difference in the prevalence of AT between the two groups among patients with AF/AT recurrence (6 of 42 [14.2%] vs 8 of 67 [11.9%], *P* = .16).

## 4 DISCUSSION

This retrospective study compared the acute and long‐term procedural outcomes of 103 patients who received a second ablation procedure using UHR‐EAM and 153 patients who received the procedure using C‐EAM. Our main results were as follows. (a) The overall one‐year AF/AT‐free survival rate was comparable between the two groups. (b) Safety outcomes were also similar between the two groups. (c) UHR‐EAM demonstrated shorter radiofrequency application time during the overall procedure, but required greater radiation exposure with longer total fluoroscopic time than C‐EAM. (d) The elimination rate of AT was significantly higher using UHR‐EAM than C‐EAM. To the best of our knowledge, this is the first clinical study to report the safety and efficacy of a repeat ablation procedure of AF using UHR‐EAM.

### 4.1 Acute procedural outcomes using UHR‐EAM

UHR‐EAM demonstrated shorter radiofrequency application time during the overall procedure. This suggests that UHR‐EAM produced more efficient ablations than C‐EAM, possibly by the precise identification of arrhythmogenic substrates such as an AT circuit and conduction gap after linear or circumferential ablation. These may be attributable to the high density of mapping points, high‐quality signal recording, accurate and flexible electrogram annotation, and efficient beat acceptance criteria.

UHR‐EAM was especially advantageous in circumferential ablation of PVs. Radiofrequency application time during re‐isolation of PVs was shorter in the UHR‐EAM group, possibly because UHR‐EAM can more precisely detect the left atrium‐PV conduction gap.^3^, ^6^ A previous study reported that the tiny electrode of the mini‐basket catheter, which clearly records the gap signal, and the excellent electrogram annotation enables visualization of the PV gap using UHR‐EAM.^3^ In addition, the efficacy of UHR‐EAM using the basket catheter for SVC isolation has also been reported.^8^, ^9^

The superior ablation outcome using UHR‐EAM was observed for the ablation of AT, including not only typical macro‐reentrant AT (perimitral and roof‐dependent) but also atypical macro‐reentrant and focal AT. UHR‐EAM enabled the efficient visualization of AT circuits, including the exact location of the slow‐conduction isthmus for macro‐reentrant AT and the earliest activation site for focal AT.^5^ In addition, UHR‐EAM allowed precise visualization of the conduction gap, markedly facilitating conduction block after linear ablation connecting two non‐conducting tissues.

### 4.2 Rhythm outcomes within 1 year after the second ablation procedure

There was no difference in the 1‐year AF/AT recurrence rates between the two groups. UHR‐EAM demonstrated some advantages of a higher elimination rate of AT than C‐EAM and low radiofrequency application time. Conversely, in the C‐EAM group, ablation catheter incorporating contact‐force monitoring sensor and magnetic location sensor possibly enabled robust ablation lesion which might improve rhythm outcomes. Similar rhythm outcomes between the two mapping system suggest that those differences would be trivial or cancel each other.

### 4.3 Safety concerns of UHR‐EAM

No severe complications developed in the 103 consecutive patients who received ablation using UHR‐EAM. Other reports have consistently shown the safety of this mapping system in the atrium and ventricle.^10^, ^11^ However, we demonstrated that UHR‐EAM required a longer fluoroscopic time than C‐EAM. Similarly, a study comparing procedural outcomes of de novo AF ablation procedures reported increased radiation exposure using UHR‐EAM.^6^ Currently, UHR‐EAM provides less reliable data on catheter location and catheter‐tissue proximity due to the lack of a magnetic technology for detecting catheter orientation and a catheter‐tissue contact force sensor. This means that operators are more dependent on fluoroscopy for determining these parameters when using UHR‐EAM than C‐EAM.

### 4.4 Clinical implications

Both of the EAMs examined in this study are safe and efficacious for repeat AF ablation. UHR‐EAM is expected to be more effective in patients with conditions complicated with AT. In addition, UHR‐EAM may improve rhythm outcomes in non‐paroxysmal AF patients.

### 4.5 Limitations

Several limitations of this study warrant mention. First, the decision to use UHR or C‐EAM was based on the discretion of the attending physicians and was not randomized. Ablation outcomes may also have been influenced by factors other than the choice of EAM, such as ablation strategy, technological developments in related equipment and operator skill. Second, the ablation strategy, especially the decision to perform additional empirical substrate modifications, was somewhat subjective. Third, the number of patients who underwent individual ablation procedures, such as ablation targeting ATs and non‐PV/SVC AF‐triggering ectopies, was too small for accurate statistical analysis. Fourth, AF recurrence after discharge was quantified on the basis of the patients' symptoms, giving rise to the possibility that asymptomatic episodes of AF might have been missed. Finally, the small sample size limits the statistical accuracy of our results. Multicenter randomized controlled trials including a sufficient number of patients are warranted.

## 5 CONCLUSION

UHR‐EAM demonstrated comparable 1‐year rhythm outcomes with efficient radiofrequency energy application to C‐EAM in patients undergoing repeat ablation for recurrent AF. Notably, UHR‐EAM produced excellent acute AT ablation outcome.

## CONFLICT OF INTEREST

The authors declare no potential conflict of interests.
